# COVID-19 vaccine acceptance among healthcare workers in China: A systematic review and meta-analysis

**DOI:** 10.1371/journal.pone.0273112

**Published:** 2022-08-12

**Authors:** Xiaoling Shui, Fang Wang, Ling Li, Qian Liang

**Affiliations:** 1 School of Nursing, Chengdu University of Traditional Chinese Medicine, Chengdu, Sichuan Province, China; 2 Nursing Department, Affiliated Hospital of Chengdu University of Traditional Chinese Medicine, Chengdu, Sichuan Province, China; The University of the West Indies, TRINIDAD AND TOBAGO

## Abstract

**Background:**

Since the successful development of Coronavirus Disease (COVID-19) vaccine, COVID-19 vaccination has been actively advocated all over the world. As the key population for COVID-19 vaccination, the acceptance of Healthcare Workers (HCWs) is not only related to their risk of contracting COVID-19 infection at work, but also affects the decision of the general population on COVID-19 vaccination. Currently, a series of observational studies have been conducted on the acceptance of COVID-19 vaccines among HCWs in China, but there are presently no all-inclusive reviews. Therefore, this paper reviewed to identify a reliable estimate of acceptance rate of COVID-19 vaccine among HCWs in China.

**Methods:**

We conducted a search on PubMed, EMbase, The Cochrane Library, Web of Science, CNKI (Chinese National Knowledge Infrastructure), Wanfang Database, CBM (Chinese Biomedical Literature Database) and VIP database (Chinese Scientific Journal Database) from January 2020 to June 2022. The quality of included articles was estimated using the Newcastle-Ottawa Quality Assessment tool suitable for cross-sectional studies and STATA 16 was used for analysis, A random-effects model was used to calculate acceptance rate for COVID-19 vaccine, as well as subgroup analysis and sensitivity analysis.

**Result:**

This review included 18 studies involving 45,760 subjects, all of which were of medium or high quality. Meta-analysis results represented that, the pooled estimated acceptance rate of COVID-19 vaccine among HCWs in China was 78% (95%CI: 73–83%), and the pooled acceptance rate in 2021 (82%, 95%CI: 78–86%) was significantly higher than that in 2020 (73%, 95%CI: 65%-81%). Subgroup analysis showed different acceptance rates for COVID-19 vaccine among HCWs with different characteristics.

**Conclusion:**

The result revealed that HCWs in China generally have a high acceptance rate of COVID-19 vaccines, but the acceptance rate varies with different characteristics of the population. Therefore, corresponding training should be carried out for HCWs with different characteristics, and they should play an exemplary and leading role in COVID-19 vaccination, so as to improve the vaccination rate of the whole population and form an immune barrier at an early date.

## Introduction

Coronavirus disease-2019 (COVID-19), a highly transmissible ailment caused by the SARS-CoV-2 virus, had become a global public distress since it was first determined in Wuhan, China, in late December 2019. World Health Organization (WHO) officially proposed to name this infectious disease as coronavirus disease 2019 (COVID-19) on 12 February 2020, and finally made the judgment that COVID-19 to have the characteristics of a global pandemic on 11 March 2020 [1]. As of 8 June 2022, COVID-19 has officially spread to 230 countries and territories, with more than 500 million confirmed cottom and 6.32 million cumulative deaths reported [2], causing great loss of financial, manpower and material resources. It estimates that almost 15 million died directly and indirectly from SARS-COV-2 in 2020 and 2021, nearly three times the reported by governments around the world [3]. We are living through an unprecedented crisis, with the COVID-19 spreading rapidly around the world in a short period of time [4] and likely to continue to have a profound impact on healthcare systems [5].

Coronavirus is a positive single-stranded RNA viruses. SARSCoV-2 belongs to the b-coronavirus subgenus, same as other RNA viruses, and SARSCoV-2 undergoes a high degree of genomic mutation during host adaptation, which poses a significant challenge to existing treatment options and prevention [6]. For purpose of better preventing novel coronavirus infections and contain the spread of the outbreak, medical workers and scientific researchers all over the world have been searching for appropriate treatment strategies, including antiviral agents, immunotherapy and vaccine [7,8]. However, herd immunity against SARS-COV-2 cannot be achieved at present due to the absence of specific medicine for COVID-19, the inability of viral infection and vaccine-induced immunity to prevent the spread of the epidemic, and the occurrence of antigenically distinct variants [9,10]. In order to prevent repeated infection in the population, safe and effective vaccine is the most efficient and reliable means to establish an immune barrier in the population.

SARS-CoV-2 vaccines have reassuring safety and is effectively in reducing deaths, symptomatic cases, severe cases and infections caused by SARS-CoV-2 worldwide [11–13]. The Advisory Committee on Immunization Practices (ACIP) proposed prioritizing healthcare workers (HCWs) vaccination in December, 2020, since HCWs have easier access to populations of COVID-19 patients during routine diagnostic and treatment activities and are at much greater risk of contracting COVID-19 than other populations [14]. There have been review representing moderate acceptance of COVID-19 vaccination among HCWs [15]. Mandatory COVID-19 Vaccination for HCWs against is a sensitive and controversial topic, with different levels of support around the world [16]. Therefore, in order to prevent the spread of COVID-19, it is critical to adopt strategies to improve the acceptance and willingness of HCWs to be vaccinated against COVID-19.

The success of a vaccine depends not only on its effectiveness, but also on the coverage of vaccination [17]. Accordingly, the main intention of this meta-analysis was to pool the willingness and acceptance to vaccinate against COVID-19 among HCWs in China, study the acceptance characteristics of COVID-19 vaccine among different types of HCWs, and provide targeted strategies for improving the promotion of COVID-19 vaccine and evidence to improve willingness to receive COVID-19 vaccine among all types of health workers.

## Materials and methods

### Protocol registration and best practice

This systematic evaluation and meta-analysis was carried out in full accordance with the guidelines of the Preferred Reporting Items for Systematic Review and Meta Analysis (PRISMA) [18] (see S1 Table), and had been registered in the Prospective Register of Systematic Reviews (PROSPERO). (ID: CRD42022337627).

### Eligibility criteria

The inclusion and exclusion criteria are developed according to the PIOT framework, which include the Population (P), Indicator (I), Outcome of interest (O), and Time (T).

### Inclusion criteria

1. The PIOT criteria are: (1) Population: HCWs from China, included clinicians, nurses, paramedics, administrators, medical examiners, medical technicians, other medical personnel and full-time staff in Center for Disease Control and Prevention (CDC), including trainee medical personnel (2) Indicator: COVID-19 vaccine (3). Results: Acceptance rate (both vaccinated and unvaccinated but willing to be vaccinated) (4). Time: during the COVID-19 pandemic.
2. Original observational studies published in English and Chinese were included.

#### Exclusion criteria

1. Studies that reported only vaccination against COVID-19
2. Reviews, comments, case reports, editorials and letters were also excluded
3. Studies in which raw data cannot be transformed
4. Acceptance rates explicitly referring to participants in other regions besides China
5. Acceptance rates explicitly referring to participants other than HCWs
6. Studies where only abstracts are available and conference literature
7. Duplicate literature was excluded and only the most complete data of the population was retained.

### Information sources and search strategies

Two investigators independently and comprehensive searched both Chinese- and English-language databases. The English-language databases were PubMed, EMbase, The Cochrane Library, and Web of Science, the Chinese- language databases were the CNKI (Chinese National Knowledge Infrastructure), Wanfang Database, CBM (Chinese Biomedical Literature Database) and VIP database (Chinese Scientific Journal Database). Omissions were prevented, there were no restrictions imposed on publication type and population, and all publication dates from January 2020 to June 2022 were suitable. Besides, the available references of included studies articles and relevant reviews were also tracked to identify gray literature. Medical Subject Headings (MeSH terms) and free text words were combined to conduct literature retrieval. The following search terms were used for each database: ‘COVID-19 Vaccines’, ‘COVID-19 Virus Vaccines’, ‘SARS-CoV-2 Vaccines’, ‘SARS2 Vaccines’, ‘2019-nCoV Vaccine’, ‘2019 Novel Coronavirus Vaccines’, ‘2019 Novel Coronavirus Vaccines’, ‘Willingness*’, ‘Hesitancy’, ‘Attitude*’, ‘Accept*’ and ‘China’. The full search string for the PubMed database is shown in the S2 Table.

### Data screening and selection

After the duplicate articles were removed, the remaining titles and/or abstracts were screened by two independent reviewers in the Endnote software, and the articles that require full text were then imported into the Zetero software and screened against pre-determined criteria. If more than one study was published using the same dataset, only the studies with the largest sample sizes were included in our review. When conflicts arised, consensus was reached through consultation with a third researcher.

### Data extraction and management

All data extraction was performed independently and simultaneously by two reviewers and then exchanged for checking to prevent data errors. If necessary, contacted the author by email for further information. We use standardized tables on the WPS Excel spreadsheet to extract data. The data extraction checklist included first author, publication year, participant group, sample size, survey period, study design, region (the area where studies were conducted), age, gender, and the outcome (acceptance rate).

### Quality assessment

Two independent reviewers used the Agency of Healthcare Research and Quality (AHRQ) assessment tool(see S2 Table) [19] to evaluate risk of bias included original studies. It contains 11 items, the answer “yes” is scored 1 point, and “no” or “not clear” is scored 0 points. Moreover, studies were classified as “high quality” (8–11), “moderate quality” (4–7) and “low quality” (0–3) based on their total scores. During the time of quality assessment, discrepancies between two independent reviewers were resolved by a third reviewers after discussion.

### Data synthesis

Pooled acceptance rate and 95% confidence intervals (CIs) were adopted to estimate the acceptance rate of Chinese HCWs and the statistical analysis STATA 16.0 was used to analyze quantitative data. The Cochrane Q test and the I^2^ value test were used to examine the heterogeneity of the studies; where <25%, 25–50%, and >50% indicated a low, moderate, and a high level heterogeneity, respectively [20]. Leave one out sensitivity analysis was performed to examine the robustness on the overall pooled estimate. Since the heterogeneity of all the Pooled results was greater than 50%, random effect model was used for analysis. Subgroup analysis were conducted based on the survey year, geographical region, gender, age, education level, occupation, monthly income, professional position, marital status, time of employment, whether to participated in quarantine or had contact with confirmed cases and whether to having chronic conditions. Potential publication bias was evaluated graphically by funnel plot and Egger test (when P < 0.05, publication bias was significant) [21].

## Results

### Search outcomes

**Fig 1:**
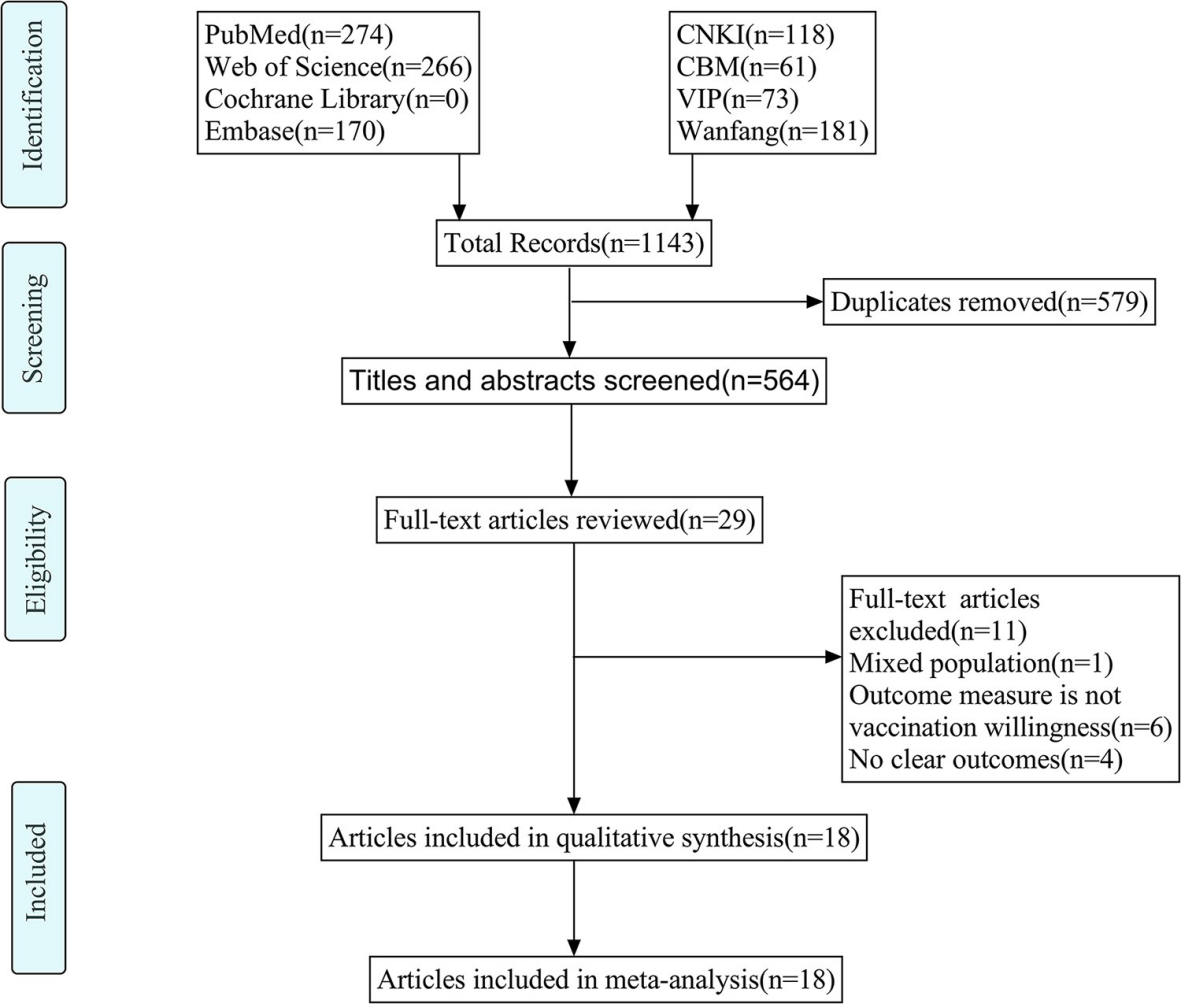
PRISMA flow diagram.

A total of 1,143 records were retrieved, and 579 records were excluded by duplicates. After excluding duplicates, 535 records were excluded by reviewing the title and abstract and 11 records were excluded based on full-text review. Finally, 18 studies were included in the final meta-analysis, including 45,760 participants [24,37]. The detailed process of selection according to the PRISMA guidelines is showed in Fig 1.

### Quality assessment

**S3 Table** represents the quality assessment using the AHRQ appraisal tool and the level of evidence for each included 18 study. In general, two studies were considered high quality [13,34], while the remaining were stated as moderate quality [22–33,35–38]. All included studies were rated "yes" in items 1, 2, and 8, "unclear" in item 4, and "no" in item 9 and 11.

### Characteristics of the studies

Cross-sectional surveys were employed in all studies. All studies used online surveys to measure acceptance rate of COVID-19 vaccines. Out of the 19 studies included, with sample sizes ranging from 416 to 11,951, included a total of 45,760 participants and were surveyed only in 2020 and 2021, with the first survey performing in February-March 2020 and the most recent in April 2021. Two studies were conducted on a scale spanning 5 provinces [13,30], two studies were conducted across 3 provinces [28,29], and four studies were conducted on whole national scale [31,32,35,38]. Two studies included nurses or practice nurses [24,37], and the remaining studies included different types of HCWs. Overall, the acceptance rate ranged from 37.62% to 95.85%. Table 1 summarises the characteristics of the included studies.

**Table 1:** Characteristics of the included studies.

Author and Year	Region	Participants	Sample size	Survey period	Age/Years	Male(%)	Acceptance rate(%)	Quality evaluation
Zhang GF,2021 [22]	Beijing	Doctors, Nurses, Paramedics	1658	Jun,2020	Majority;30-39(39.75%)	21.29%	68.40%	Moderate
Yu,2022 [23]	Hunan, Huaihua	Clinicians, Nurses, Technicians, Public health physicians, Administrators, Ancillary staff	3958	Not stated	Majority;20-29(37.49%)	23.37%	85.70%	Moderate
Liu,2022 [24]	Anhui	Nursing trainees	551	Dec,2020	18.34±1.31	11.07%	57.53%	Moderate
Luo,2021 [25]	Sichuan	CDC staff	551	Jan,2021	Majority;30-39(29.4%)	41.70%	84.57%	Moderate
Kong,2021 [13]	Shanxi, Beijing, Shandong, Hubei, Sichuan	Clinicians, Nurses, Technicians, Administrators, Medical examiners, CDC staff	9345	Nov,2020	Majority;30-39(35.23%)	27.33%	70.82%	High
Cheng,2022 [26]	Yunnan	CDC staff	416	Dec,2020	Majority;30-50(48.08%)	41.35%	83.65%	Moderate
Zhang HJ,2021 [27]	Zhejiang	Healthcare workers and CDC staff	756	Sept,2020	Majority;31-40(36.11%)	33.07%	70.11%	Moderate
Shi,2022 [28]	Shanghai, Wuhan, Lanzhou	Healthcare workers	627	Jun,2020	Majority;30-39(57.1%)	49.76%	95.85%	Moderate
Hao,2022 [29]	Inner Mongolia, Beijing, Hebei	Full-time healthcare workers	621	Apr,2021	Majority;31-50(57.1%)	28.50%	68.28%	Moderate
Wang H,2022 [30]	Henan, Sichuan, Shandong, Guangdong, Inner Mongolia, Xinjiang, Liaoning	Healthcare workers, Administrators excluded	2681	Jan-May,2021	Majority;25-34(37.22%)	27.94%	82.54%	Moderate
Wang MW,2021 [31]	33 provinces	Healthcare workers	1329	Jan,2021	Majority;18-24(29.0%)	35.40%	76.98%	Moderate
Ye,2021 [32]	21 provinces	Doctors, Nurses	2156	Feb,2021	32.91±8.29	12.10%	87.94%	Moderate
Li,2021 [33]	whole China	Doctors, Nurses, Ancillary staff, and others	1779	Jan-Feb,2021	Majority;18-29(41.7%)	11.80%	93.87%	Moderate
Sun,2021 [34]	Sichuan, Chengdu	Healthcare workers	505	Jan,2021	32.35±8.98	22.57%	76.63%	High
Wang C,2021 [35]	31 provinces	Healthcare workers	2386	Jan,2021	Majority;30-39(33.1%)	37.05%	80.85%	Moderate
Wang J,2021 [36]	Zhejiang	Doctors, Health technicians, Nurses and others	3634	Sept,2020	Majority;<50(88.11%)	22.56%	79.09%	Moderate
Wang KL,2020 [37]	Hong Kong	Nurses	856	Feb-Mar,2020	Majority;30-39(31.1%)	11.80%	37.62%	Moderate
Huang,2021 [38]	30 provinces	doctors, nurses, and other medical professionals (medical and nursing trainees, technicians, and clinical pharmacists) working in ICUs	11951	Mar-Apr,2021	Majority;31-40(45.8%)	17.70%	84.65%	Moderate

### Synthesis of results

**Fig 2:**
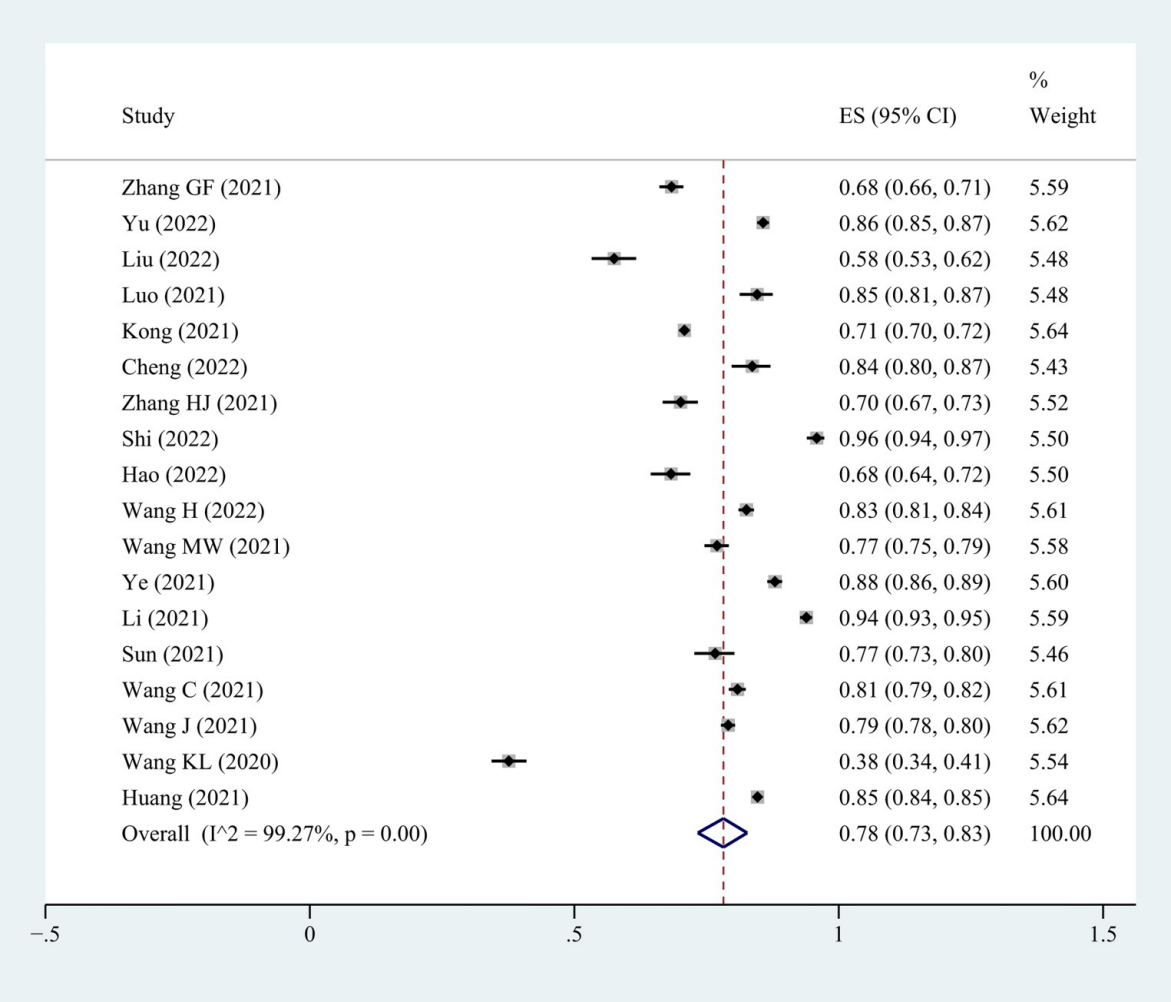
Forest plot showing COVID -19 vaccine acceptance rate among HCWs in China.

Based on the random-effects model, pooled COVID-19 vaccine acceptance rate and 95% confidence interval among HCWs in China was 78% (95% CI: 73%-83%). However, There was strong heterogeneity among these studies (I^2^ = 99.27%, p<0.001) (Fig 2).

### Sensitivity analysis

**Fig 3:**
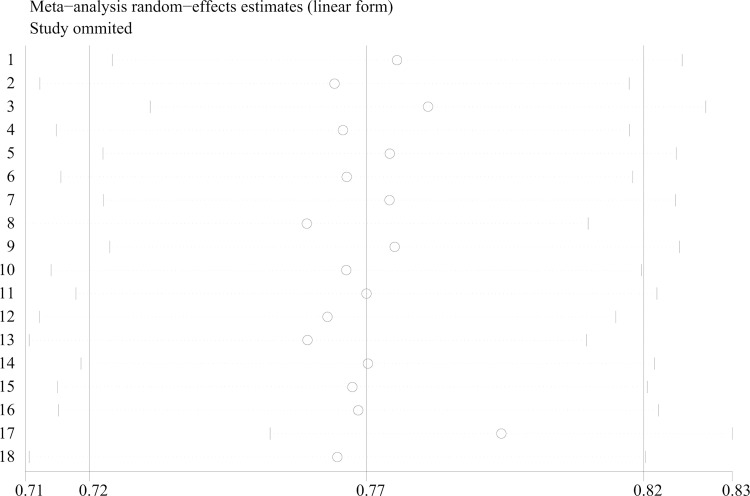
Sensitivity analysis for COVID-19 vaccine acceptance rate among HCWs in China.

Sensitivity analysis of the total acceptance rate was performed by eliminating individual studies one by one. The results showed that no single study affected the overall estimated acceptance rate. After excluding any included studies, there was little change in estimated acceptance rate, suggesting that the robustness of this analysis. (S4 Table and Fig 3).

### Subgroup analysis

As shown in **Table 2**, subgroup analysis was based on survey year, the pooled estimated COVID-19 vaccine acceptance rate (73%, 95% CI: 65%-81%) in 2020 was significantly lower than that in 2021 (82%, CI: 78%-86%). Based on gender, the estimated pooled COVID-19 vaccine acceptance for male HCWs (83%, 95% CI: 78%-88%) was slightly higher than that for the female HCWs (80%, 95% CI: 74%-85%). Based on education level, the higher the education level, the lower the estimated pooled acceptance rate of COVID-19 vaccine. The acceptance rate of college degree or below was 84% (CI: 79%-89%), bachelor degree was 82% (CI: 78%-86%), and undergraduate degree or above was 79% (CI: 72%-86%). Based on occupation, the pooled estimated COVID-19 acceptance rate for nurses (76%, 95% CI: 68%-83%) was significantly lower than HCWs in other occupation. Based on Monthly income, The acceptance rate of HCWs with monthly income over 10000 yuan was the lowest (78%, 95% CI: 55%-94%). Based on region, the acceptance rate in Northern China was the lowest (79%, 95% CI: 77%-80%). the acceptance rate in Southern China was the highest (87%, 95% CI: 84%-90%). Based on professional position, the pooled estimated COVID-19 acceptance rate for HCWs with senior titles (85%, 95% CI: 78%-91%) was higher than HCWs with other professional titles. Based on marital status, widowed or divorced HCWs (70%, 95% CI: 38%-95%) had lower acceptance rate than married or unmarried HCWs. Moreover, acceptance of COVID-19 vaccine was low among HCWs with chronic conditions (75%, 95% CI: 57%-90%) and those who had not been participated in quarantine or contact with confirmed cases (78%, 95% CI: 69%-86%). Based on time of employment, HCWs with time of employment less than 5 years (83%, 95% CI: 78%-87%) and those with time of employment more than 20 years (83%, 95% CI: 79%-87%) had lower acceptance of COVID-19 vaccine.

**Table 2:** Subgroup analysis based on the characteristics of the included subjects.

Study characteristics	No. of studies	Acceptance rate (%) (95% CI)	I^2^	p-value
**Survey year**				
2020	9	73(65–81)	99.19%	<0.001
2021	8	82(78–86)	98.17%	<0.001
**gender**				
male	16	83(78–88)	97.37%	<0.001
female	16	80(74–85)	99.19%	<0.001
**age(year)**				
<30	11	81(75–86)	97.92%	<0.001
30–39	8	73(60–85)	99.17%	<0.001
40–49	8	81(69–90)	98.38%	<0.001
≥50	11	86(76–93)	96.68%	<0.001
**Education Level**				
College degree or below	11	84(79–89)	97.54%	<0.001
Bachelor degree	11	82(78–86)	98.25%	<0.001
Postgraduate and above	11	79(72–86)	95.46%	<0.001
**Occupation**				
doctors	9	85(79–90)	96.96%	<0.001
Nurses	10	76(68–83)	99.24%	<0.001
others	10	84(82–87)	70.57%	<0.001
**Monthly income(yuan)**				
≤5000	5	85(75–94)	97.60%	<0.001
5001–1000	5	84(73–92)	98.43%	<0.001
>10000	4	78(55–94)	98.79%	<0.001
**region**				
Eastern	6	82(69–92)	99.31%	<0.001
Southern	4	87(84–90)	89.10%	<0.001
Western	7	80(68–90)	99.20%	<0.001
Northern	2	79(77–80)	0%	<0.001
**Professional position**				
Junior or no	6	83(78–88)	98.63%	<0.001
Middle	6	83(77–89)	97.57%	<0.001
Senior	6	85(78–91)	94.62%	<0.001
**Marital status**				
Married	5	87(82–91)	95.33%	<0.001
Unmarried	5	87(80–93)	94.54%	<0.001
Others (Widowed or divorced)	4	70(38–95)	89.02%	<0.001
**Having chronic conditions**				
Yes	5	75(57–90)	98.06%	<0.001
No	5	80(64–93)	99.68%	<0.001
**Time of employment(year)**				
≤5	5	83(78–87)	88.94%	<0.001
6–10	5	85(80–89)	88.36%	<0.001
11–20	4	85(79–89)	85.71%	<0.001
>20	4	83(79–87)	77.17%	<0.001
**Participated in quarantine or had contact with confirmed cases**				
Yes	8	83(75–90)	98.82%	<0.001
No	8	78(69–86)	99.31%	<0.001

### Publication bias analysis

**Fig 4:**
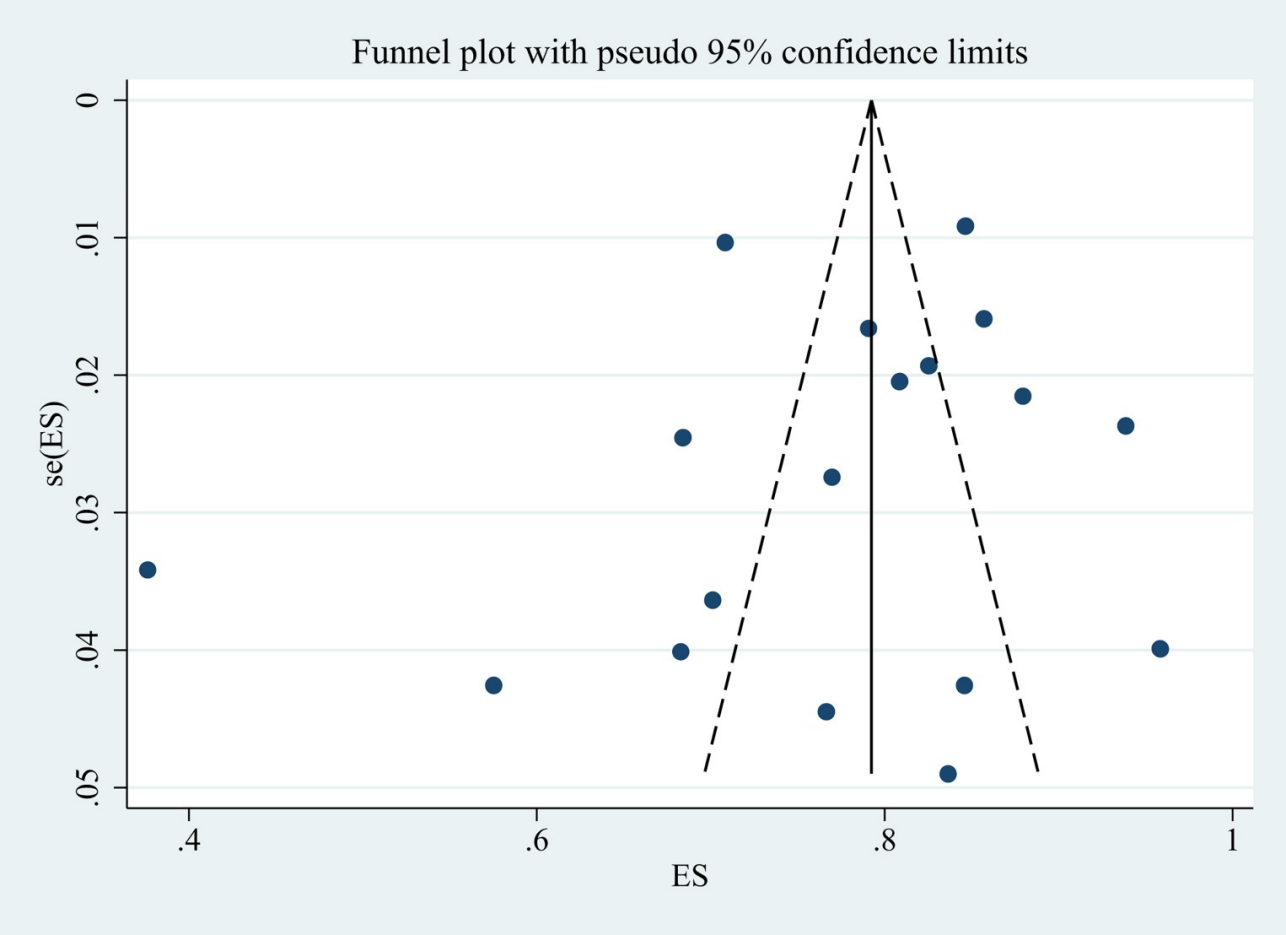
Funnel plot.

There was no obvious potential publication bias after symmetrical inspection using the funnel plot (**Fig 4**) and Egger’s regression test (t = -0.74, P = 0.469).

## Discussion

This systematic review aimed to comprehensively pool the acceptance rate of COVID-19 vaccination among HCWs in China, to provide evidence for future improvements in vaccination implementation strategies. This systematic review and meta-analysis showed a pooled overall acceptance rate (78%) for COVID-19 vaccination among HCWs in China. But this result was lower than the acceptance rate in three previous national cross-sectional studies with more than 2,000 participants (87.94% [32], 80.85% [35], and 84.65% [38], respectively) and slightly higher than the result in a national study [31] with 1,329 participants, which indicated an overall acceptance rate of 76.98%. In addition to the small sample effect, another possible contributor to this finding was that in the latter study, the addition of attitudes of hesitation to the questionnaire led to a lower overall acceptance rate. However, this result was significantly higher than the pooled acceptance rate of COVID-19 vaccine among HCWs around the world (55%) reported in a meta-analysis [15].

Firstly, we found that vaccination intentions did vary by survey time, with vaccination intentions significantly higher in 2021 than in 2020 [39]. One possible explanation for this was that the amount of information related to COVID-19 and COVID-19 vaccines had been tremendous in recent years, and had increased at an unprecedented pace. As passage of time, scientific evidence about COVID-19 vaccine had become more comprehensive, and HCWs had become more aware of its safety and effectiveness [40]. In addition, The National Health Commission of China’s policy of voluntary and free vaccination with informed consent of all adults, issued in January 2021, also played a big role. Secondly, the subgroup analysis between gender indicated that male health workers were more likely than females to receive COVID-19 vaccines [41–48]. The likely reason was that COVID-19 severity and mortality were increased in male compared to female [49]. It may also be related to women’s special conditions, such as menstruation, pregnancy and lactation, which affect women’s willingness to get vaccinated against COVID-19. Moreover, it was worth noting that the results of this study showed that the willingness of HCWs to vaccinate against COVID-19 declined with the improvement of educational level, which was contrary to the results in Africa [50], suggesting that educational level played different roles in the willingness of different populations to vaccinate against COVID-19. Those with higher education levels were likely to receive more adverse information such as rumors about vaccines. We also found relatively low acceptance rate of COVID-19 vaccine among nurses compared to doctors and other HCWs, similar to a survey conducted in Kuwait [51] and in the India[44,46–48,52]. The fact that nurses have a negative attitude towards COVID-19 vaccine was of concern since they were the most vulnerable subgroup to be infected and it is important to improve the levels of trust in the efficacy and safety of COVID-19 vaccines in this subgroup. In this study, there were also differences in vaccination intentions among HCWs in different regions, with southern China having the highest vaccination rates (87%), and a global review of HCWs also confirmed differences in vaccination intentions among HCWs in different regions [15]. Better publicity and health education should be carried out for HCWs in regions with low vaccination intentions to improve their acceptance of COVID-19 vaccine. Finally, HCWs who participated in quarantine or had been in contact with confirmed cases had a stronger incentive to get vaccinated against COVID-19, because they believe they were more vulnerable to infection, which also affects the general population. A cross-sectional study in Iraqi showed that HCWs believe they were more vulnerable to COVID-19 and were more likely to be vaccinated against the disease than the general population. Targeted dissemination of COVID-19 knowledge was needed to improve the willingness of HCWs to receive COVID-19 vaccine and eliminate vaccine hesitancy among HCWs. At the same time, regarding adverse events in vaccine development and use, government departments and institutions should release authoritative, transparent and objective information in a timely manner to reduce vaccine hesitation caused by canard of Internet.

### Strengths and limitations

#### The advantages of this study

First, in order to avoid repeated accidental duplication of reviews, reduce potential bias, and improve transparency, this study conducted a systematic review of prospective registries [53]. Therefore, to the authors’ knowledge, this is the first systematic review and meta-analysis of COVID-19 acceptance rates in China. Second, this review conducted a comprehensive literature search of eight databases, including published literature in Chinese and English, and tracked references to related studies to prevent omissions. In addition, the review used well-validated meta-analysis processes and models that fully conformed to international standards, and conducted sensitivity analyses to test the pooled results of the meta-analyses. Finally, the literature included in this review covered the majority of regions in China and was well representative.

### Limitations of this study

First, there was significant heterogeneity among all articles, and only two articles were rated as high quality. First of all, the limited number of studies included in this meta-analysis may lead to bias in the inferred and summarized results, which reduced the reliability of research results. Secondly, all studies were conducted in the form of network survey, which may cause deviation in the selection of participants. Finally, although subgroup analysis was conducted according to population characteristics, the heterogeneity of each subgroup analysis was still high, which may be affected by a variety of factors such as survey tools. However, due to the limited available evidence, it could not be analyzed one by one.

## Conclusion

In summary, pooled evidence showed that the estimated COVID-19 vaccine acceptance rate of HCWs in China is 78%; Among them, the acceptance rate of HCWs with different characteristics varies, and the pooled acceptance rate in 2021 was significantly higher than that in 2020. Therefore, based on these results, we should strengthen publicity on the safety and effectiveness of vaccines, improve HCWs ’ understanding of COVID-19 vaccines, and enhance their confidence in vaccines. The publicity of contraindications for COVID-19 vaccination should be strengthened to eliminate HCWs ’ concerns about COVID-19 vaccine and improve their willingness to be vaccinated.

## Supporting information

S1 Table PRISMA 2009 checklist. (DOC)

S2 Table Search string for PubMed. (DOCX)

S3 Table ARHQ methodology checklist for cross-sectional/prevalence study. (DOCX)

S4 Table Quality evaluation of the 18 studies included in the meta-analysis. (DOCX)

S5 Table Leave one out sensitivity analysis of pooled COVID-19 vaccine acceptance rate among HCWs in China. (DOCX)
