# Supplementary material for: COVID-19 vaccine acceptance among healthcare workers in China: A systematic review and meta-analysis
Source: PLoS One. 2022 Aug 12;17(8):e0273112. doi: 10.1371/journal.pone.0273112 (PMC9374244; doi:10.1371/journal.pone.0273112)
Supplement: S2 Table — (DOCX) [file pone.0273112.s002.docx]

| S1 Table: Search string for PubMed | |
| --- | --- |
| Search# | Search Term |
| 1 | "COVID-19 Vaccines"[Mesh] |
| 2 | (COVID 19 Vaccines[Title/Abstract]) OR (Vaccines, COVID-19[Title/Abstract]) OR (COVID-19 Virus Vaccines[Title/Abstract]) OR (COVID 19 Virus Vaccines[Title/Abstract]) OR (Vaccines, COVID-19 Virus[Title/Abstract]) OR (Virus Vaccines, COVID-19[Title/Abstract]) OR (COVID-19 Virus Vaccine[Title/Abstract]) OR (COVID 19 Virus Vaccine[Title/Abstract]) OR (Vaccine, COVID-19 Virus[Title/Abstract]) OR (Virus Vaccine, COVID-19[Title/Abstract])) OR (COVID19 Virus Vaccines[Title/Abstract])) OR (Vaccines, COVID19 Virus[Title/Abstract]) OR (Virus Vaccines, COVID19[Title/Abstract]) OR (COVID19 Virus Vaccine[Title/Abstract]) OR (Vaccine, COVID19 Virus[Title/Abstract]) OR (Virus Vaccine, COVID19[Title/Abstract]) OR (COVID19 Vaccines[Title/Abstract]) OR (Vaccines, COVID19[Title/Abstract]) OR (COVID19 Vaccine[Title/Abstract]) OR (Vaccine, COVID19[Title/Abstract]) OR (SARS-CoV-2 Vaccines[Title/Abstract]) OR (SARS CoV 2 Vaccines[Title/Abstract]) OR (Vaccines, SARS-CoV-2[Title/Abstract]) OR (SARS-CoV-2 Vaccine[Title/Abstract]) OR (SARS CoV 2 Vaccine[Title/Abstract]) OR (Vaccine, SARS-CoV-2[Title/Abstract]) OR (SARS2 Vaccines[Title/Abstract]) OR (Vaccines, SARS2[Title/Abstract]) OR (SARS2 Vaccine[Title/Abstract]) OR (Vaccine, SARS2[Title/Abstract]) OR (Coronavirus Disease 2019 Vaccines[Title/Abstract]) OR (Coronavirus Disease 2019 Vaccine[Title/Abstract]) OR (Coronavirus Disease 2019 Virus Vaccine[Title/Abstract]) OR (Coronavirus Disease 2019 Virus Vaccines[Title/Abstract]) OR (Coronavirus Disease-19 Vaccines[Title/Abstract]) OR (Coronavirus Disease 19 Vaccines[Title/Abstract]) OR (Vaccines, Coronavirus Disease-19[Title/Abstract]) OR (Coronavirus Disease-19 Vaccine[Title/Abstract]) OR (Coronavirus Disease 19 Vaccine[Title/Abstract]) OR (Vaccine, Coronavirus Disease-19[Title/Abstract]) OR (COVID 19 Vaccine[Title/Abstract]) OR (Vaccine, COVID 19[Title/Abstract]) OR (2019-nCoV Vaccine[Title/Abstract]) OR (2019 nCoV Vaccine[Title/Abstract]) OR (Vaccine, 2019-nCoV[Title/Abstract]) OR (2019 Novel Coronavirus Vaccines[Title/Abstract]) OR (2019 Novel Coronavirus Vaccine[Title/Abstract]) OR (2019-nCoV Vaccines[Title/Abstract]) OR (2019 nCoV Vaccines[Title/Abstract]) OR (Vaccines, 2019-nCoV[Title/Abstract]) OR (COVID-19 Vaccine[Title/Abstract]) OR (Vaccine, COVID-19[Title/Abstract]) OR (SARS Coronavirus 2 Vaccines[Title/Abstract]) |
| 3 | (Willingness*[Title/Abstract]) OR (Hesitancy[Title/Abstract]) OR (Accept*[Title/Abstract]) OR (Attitude*[Title/Abstract]) |
| 4 | China[Title/Abstract] |
| 5 | #1 OR #2 |
| 6 | #3 AND #4 AND #5 |
