# Supplementary material for: COVID-19 vaccine acceptance among healthcare workers in China: A systematic review and meta-analysis
Source: PLoS One. 2022 Aug 12;17(8):e0273112. doi: 10.1371/journal.pone.0273112 (PMC9374244; doi:10.1371/journal.pone.0273112)
Supplement: S3 Table — (DOCX) [file pone.0273112.s003.docx]

| **S3 Table.ARHQ Methodology Checklist for Cross-Sectional/Prevalence Study** |
| --- |
| Website: http://www.ncbi.nlm.nih.gov/books/NBK35156/ |
| ① Define the source of information (survey, record, review) |
| ②List inclusion and exclusion criteria for exposed and unexposed subjects (cases and controls) or refer to previous publications |
| ③ Indicate time period used for identifying patients |
| ④Indicate whether subjects were consecutive if not population-based |
| ⑤ Indicate if evaluators of subjective components of study were blind to other aspects of the status of the participants |
| ⑥ Describe any assessments undertaken for quality assurance purposes (e.g., test-retest of primary outcome measurements) |
| ⑦ Explain any exclusions of data from analysis |
| ⑧ Describe how confounding was assessed and-or controlled |
| ⑨ If applicable, explain how missing data were handled in the analysis |
| ⑩ Summarize patient response rates and completeness of data collection |
| ⑪ Clarify what follow up, if any, was expected and the percentage of patients for which incomplete data or follow-up was obtained |
