# Supplementary material for: COVID-19 vaccine acceptance among healthcare workers in China: A systematic review and meta-analysis
Source: PLoS One. 2022 Aug 12;17(8):e0273112. doi: 10.1371/journal.pone.0273112 (PMC9374244; doi:10.1371/journal.pone.0273112)
Supplement: S4 Table — (DOCX) [file pone.0273112.s004.docx]

| **S4 Table. Quality evaluation of the 18 studies included in the meta-analysis** | | | | | | | | | | | | |
| --- | --- | --- | --- | --- | --- | --- | --- | --- | --- | --- | --- | --- |
| **Studies** | **Items** | | | | | | | |  |  |  | **Score of Quality** |
|  | 1 | 2 | 3 | 4 | 5 | 6 | 7 | 8 | 9 | 10 | 11 |  |
| Zhang GF,2021 | Y | Y | Y | N | U | N | Y | Y | N | Y | N | 6 |
| Yu,2022 | Y | Y | N | N | U | N | N | Y | N | Y | N | 5 |
| Liu,2022 | Y | Y | Y | N | U | N | N | Y | N | Y | N | 5 |
| Luo,2021 | Y | Y | Y | Y | U | N | Y | Y | N | Y | N | 7 |
| Kong,2021 | Y | Y | Y | Y | U | Y | Y | Y | N | Y | N | 8 |
| Cheng,2022 | Y | Y | Y | N | U | N | Y | Y | N | Y | N | 6 |
| Zhang HJ,2021 | Y | Y | Y | N | U | N | N | Y | N | N | N | 4 |
| Shi,2022 | Y | Y | Y | N | U | N | Y | Y | N | N | N | 5 |
| Hao,2022 | Y | Y | Y | N | U | N | Y | Y | N | Y | N | 6 |
| Wang H,2022 | Y | Y | Y | N | U | N | Y | Y | N | Y | N | 6 |
| Wang MW,2021 | Y | Y | Y | N | U | N | Y | Y | N | Y | N | 6 |
| Ye,2021 | Y | Y | Y | N | U | Y | Y | Y | N | Y | N | 7 |
| Li,2021 | Y | Y | Y | N | U | Y | Y | Y | N | Y | N | 7 |
| Sun,2021 | Y | Y | Y | Y | U | Y | Y | Y | N | Y | N | 8 |
| Wang C,2021 | Y | Y | Y | N | U | N | Y | Y | N | Y | N | 6 |
| Wang J,2021 | Y | Y | Y | Y | U | N | Y | Y | N | Y | N | 7 |
| Wang KL,2020 | Y | Y | Y | Y | U | N | Y | Y | N | Y | N | 7 |
| Huang,2021 | Y | Y | Y | Y | U | N | Y | Y | N | Y | N | 7 |

Y, yes; N, no; U, unclear; An item would be scored ‘0’ if it was answered ‘NO’ or ‘UNCLEAR’; if it was answered ‘YES’, then the item scored ‘1’. Article quality was assessed as follows: low quality = 0–3; moderate quality = 4–7; high quality = 8–11.
