# Supplementary material for: COVID-19 vaccine acceptance among healthcare workers in China: A systematic review and meta-analysis
Source: PLoS One. 2022 Aug 12;17(8):e0273112. doi: 10.1371/journal.pone.0273112 (PMC9374244; doi:10.1371/journal.pone.0273112)
Supplement: S5 Table — (DOCX) [file pone.0273112.s005.docx]

| **S5 Table. Leave one out sensitivity analysis of pooled COVID-19 vaccine acceptance rate among HCWs in China.** | | | | |
| --- | --- | --- | --- | --- |
| no. | **Author and Year** | Pooled estimate | 95% CI | I² |
| 1 | Zhang GF,2021 | 79 | 74-83 | 99.28 |
| 2 | Yu,2022 | 78 | 73-83 | 99.29 |
| 3 | Liu,2022 | 79 | 74-84 | 99.28 |
| 4 | Luo,2021 | 78 | 73-82 | 99.28 |
| 5 | Kong,2021 | 79 | 74-84 | 99.12 |
| 6 | Cheng,2022 | 78 | 73-83 | 99.32 |
| 7 | Zhang HJ,2021 | 79 | 74-83 | 99.31 |
| 8 | Shi,2022 | 77 | 72-81 | 99.26 |
| 9 | Hao,2022 | 78 | 74-83 | 99.30 |
| 10 | Wang H,2022 | 78 | 73-83 | 99.31 |
| 11 | Wang MW,2021 | 78 | 73-83 | 99.32 |
| 12 | Ye,2021 | 78 | 72-82 | 99.28 |
| 13 | Li,2021 | 77 | 72-82 | 99.20 |
| 14 | Sun,2021 | 78 | 73-83 | 99.32 |
| 15 | Wang C,2021 | 78 | 73-83 | 99.32 |
| 16 | Wang J,2021 | 78 | 73-83 | 99.32 |
| 17 | Wang KL,2020 | 80 | 76-84 | 99.03 |
| 18 | Huang,2021 | 78 | 72-83 | 99.24 |
